# 3D Cell Cultures: Evolution of an Ancient Tool for New Applications

**DOI:** 10.3389/fphys.2022.836480

**Published:** 2022-07-22

**Authors:** Andrea Cacciamali, Riccardo Villa, Silvia Dotti

**Affiliations:** Istituto Zooprofilattico Sperimentale della Lombardia e dell’Emilia-Romagna, Laboratorio di Controllo di Prodotti Biologici. Centro di Referenza Nazionale per i Metodi Alternativi, Benessere e Cura degli Animali da Laboratorio, Brescia, Italy

**Keywords:** *in vitro*, 3D cell culture, bioreactors, organ-on-a-chip, organoids

## Abstract

Recently, research is undergoing a drastic change in the application of the animal model as a unique investigation strategy, considering an alternative approach for the development of science for the future. Although conventional monolayer cell cultures represent an established and widely used *in vitro* method, the lack of tissue architecture and the complexity of such a model fails to inform true biological processes *in vivo*. Recent advances in cell culture techniques have revolutionized *in vitro* culture tools for biomedical research by creating powerful three-dimensional (3D) models to recapitulate cell heterogeneity, structure and functions of primary tissues. These models also bridge the gap between traditional two-dimensional (2D) single-layer cultures and animal models. 3D culture systems allow researchers to recreate human organs and diseases in one dish and thus holds great promise for many applications such as regenerative medicine, drug discovery, precision medicine, and cancer research, and gene expression studies. Bioengineering has made an important contribution in the context of 3D systems using scaffolds that help mimic the microenvironments in which cells naturally reside, supporting the mechanical, physical and biochemical requirements for cellular growth and function. We therefore speak of models based on organoids, bioreactors, organ-on-a-chip up to bioprinting and each of these systems provides its own advantages and applications. All of these techniques prove to be excellent candidates for the development of alternative methods for animal testing, as well as revolutionizing cell culture technology. 3D systems will therefore be able to provide new ideas for the study of cellular interactions both in basic and more specialized research, in compliance with the 3R principle. In this review, we provide a comparison of 2D cell culture with 3D cell culture, provide details of some of the different 3D culture techniques currently available by discussing their strengths as well as their potential applications.

## Introduction

The vision of research in the 21st century is undergoing a drastic paradigm shift in the application of the animal model as a unique strategy of analysis and investigation. It has been a few decades since the 3Rs principles were devised by Russell and Burch in 1959 (Russel and Burch, 1959). This so different approach to intending an experimental design, enforced the scientific community to evaluate a new and alternative path for the development of science for the future.

Over time, this principle has been integrated with the concept of non-animal based methods, and it was included in the European Directive n. 63/2010 (EU 63/2010). In particular, the European regulatory decision to ban the animal model for research in cosmetics, forced the developer to set up and validate alternative methods in order to continue the production of new active principle and final products. This strong effort allowed us to change a consolidated, but outdated way of thinking focused on *in vivo*, with innovative and alternative methods.

In this framework, different actors are involved: stakeholders, researchers, regulatory, etc., and each one has a specific point of view in applying non-animal methods as an alternative to the *in vivo* model. One of the crucial and shared concerns among all the parts is the potential pain and suffering caused to the laboratory animals during the experiments. This aspect has been the keystone that allowed the replacement of *in vivo* with *in vitro* assays in the cosmetic field (Duval et al., 2017). In this context, several non-animal based approaches have been studied and developed. In particular, cell cultures represented, as of now, an example of not only a consolidated *in vitro* method, but also a springboard for novel and more advanced approaches linked to new biotechnologies.

The continuous evolution of the 3D model linked to cell cultures, makes it difficult to have a clear idea of the state of the art, either in the knowledge or in the dissemination of the use of this new model. Two-dimensional (2D) cell culture has represented the main method of *in vitro* research for a long time and several diagnostic, pharmaceutical and toxicological advances have been developed thanks to cell culture assays. This model has many limitations that can be overcome by the next generation cell culture approach; the three-dimensional model has shown improvements in studies aimed at morphology, proliferation, differentiation, response to stimuli, and drug metabolism (Duval et al., 2017). All this is made possible by the ability of 3D cultures to model a cell *in vivo* while it is cultured *in vitro* (Ravi et al., 2015). 3D cell culture has many applications such as cancer research, stem cell research, drug discovery, and research related to different types of diseases.

This novel approach makes it possible to bridge the gap between *in vitro* and *in vivo* model, moving through new and alternative biotechnologies, such as the use of hydrogels able to mimic the extracellular matrix (ECM) behavior and growing factors activity.

Bioreactors represent another strategic bioengineering innovation in the study of metabolic processes and represent a challenging tool in the field of regenerative medicine, especially for the development of new models either for clinical trials or for based/translational research (Wendt et al., 2009).

Organ-on-a-chips are a microfluidic culture devices for culturing and observing living cells. They play a strategic role not only in the development of the organ, but in mimicking the metabolic and physiological behavior of an *in vivo* system. In this point of view, organs-on-chips can improve knowledge regarding the cross talk between organs, especially the secretory functions. In the future, they will be the strength of drug research and development, enabling a drastic reduction in the use of laboratory animals (Bhatia and Ingber, 2014).

Organoids are the new frontier applied to Pluripotent Stem Cells (PSCs) or Adult Stem Cells (AdSCs) from which they are derived. The observation and study of organoids can supply information regarding the process of organ development and regeneration, mimicking the animal model. The culture of tumor models by organoids derived from tissue culture cells of patients can improve the research linked to drug discovery and application (Drost and Clevers, 2018).

Bioprinting is a device that can provide bioengineering support to some cell culture tolls described above. Briefly, this technology is able to print a biological entity using cells as bioink or other materials to create scaffolds for 3D assay (Arslan-Yildiz et al., 2016; Matai et al., 2020). The potential applications of this device in regenerative medicine and in research are still under study. In fact, the improvements linked to bioink and new approaches methodologies (NAMs) represent the challenges for the next generation of medicine.

As above mentioned, it seems important to supply a useful review to take stock of what is known about the use of cell cultures, the limits and the potential new applications in the scenario of alternative methods to *in vivo* model. In this regard, the aim of this paper is to focus the attention on the different facets of the 3D model: bioreactors, organs-on-chips, organoids, bioprinting, and the numerous likelihood of using these biological structures in different medical areas.

### From two to three Dimensions

Since the first approach to the isolation and amplification of cell culture, it was immediately clear that this biological assay would be a promising and useful tool in the scientific field (Yao & Asayama, 2017).

In the history of cell culture, there are some milestones that highlight the progress in the application of this *in vitro* method. One of the main limits in the use of cells is represented by the finished number of amplifications linked to isolated healthy somatic cells. For this reason, the discovery made by Earle W.R. in 1940 (Earle et al., 1943), to create immortal mouse fibroblasts using carcinogens, represented enormous progress in the possibility of application of cell culture.

However, the real milestone in the immortalization process of cell culture was the isolation, in 1951, of HeLa cells from a uterine cervical cancer human tissue (Gey et al., 1952). The use of HeLa cells contributes to spread and improves the application of *in vitro* methods world-wide; in fact, the possibility to use an immortalized and homogeneous cell culture, guarantees standardized and reproducible results.

The progress in the use of this biological method was strongly linked to the development of culture media and the other supplements necessary for the growth of cells (Lewis, 1922).

**FIGURE 1:**
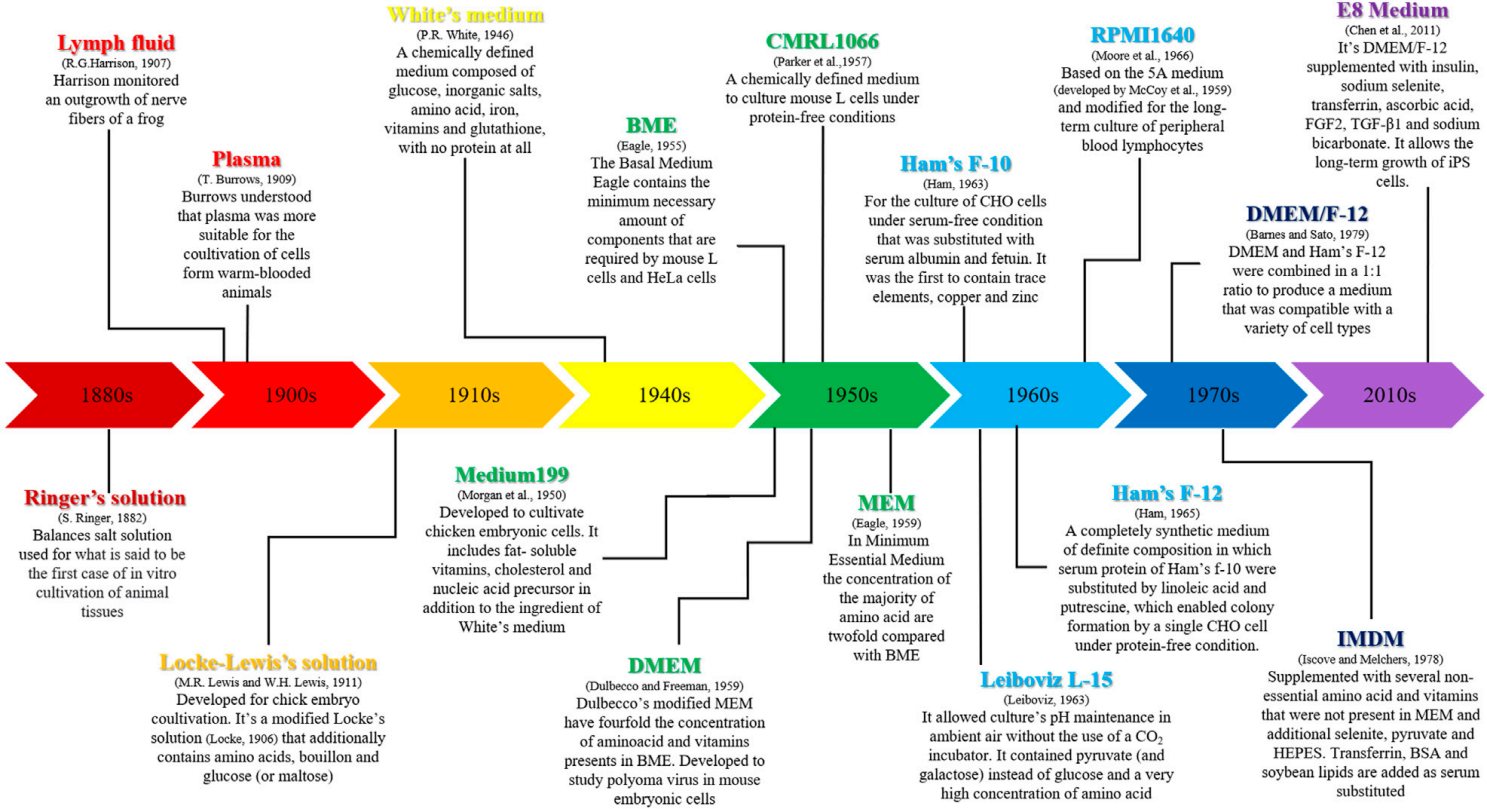
Development and applications of the main media used for cell cultures.

Since 1911, several studies have been performed in order to improve knowledge regarding amino acids, vitamins and other supplements necessary to growth of cells (Baker, 1929). In Figure 1 there is a description of the main steps linked to the discovery of media for cell culture and their applications.

The progress of biotechnology, materials (natural and artificial), media and, in general, the consumables used *in vitro* methods, have permitted to develop new approaches to cell culture applications. The passage from two to three dimensions has marked a turning point in the potential approach and use of this biological method as an alternative to the *in vivo* model (Bédard et al., 2020).

In a very schematic manner, 2D cell culture grows in a static, rigid, but standardized monolayer, otherwise the third dimension permits growth as aggregates or spheroids. The traditional monolayer is composed of proliferating cells whose morphology does not represent the normal and real aspect as *in vivo*. For this reason, several cellular processes, such as proliferation, differentiation or apoptosis can be abnormal or not completely true (Edmondson et al., 2014). In a 3D system, cells grow in a more harmonic and similar to the *in vivo* model, either if cells use a scaffold or a free-matrix approach. As described in several papers (Antoni et al., 2015; Duval et al., 2017; Terrel et al., 2020), it should consider different aspects in order to choose the better *in vitro* model to use.

In the following chapters, it will be described the most promising 3D systems with different applications and function.

### 3D Model

As a brief introduction to the 3D model, it could be useful to identify the main differences in this type of application.

Cell culture can grow with a tridimensional structure using different methods/support. As described by Bédard et al. (Bédard et al., 2020), there is the possibility to use scaffold system: synthetic (ceramics, metals or polymers) or natural (polysaccharides, proteins, ECM-derived, acellular matrix) and hydrogel. In the alternative, it is possible to adapt the cells to a scaffold free growth, as in the case of spheroids, organoids or self-assembly.

As mentioned before, the 3D cell culture model presents several aspects and applications. Since the first discovery of the possibility of reproducing a specific environment mimicking the *in vivo* model, the 3D approach represents a fabulous innovation in the panorama of alternative methods.

A 3D cell culture is defined “*by a cell culture that can mimic a living organ’s organization and microarchitecture*” (Huh et al., 2011). The routinely use of 2D platforms has permitted a robust and consolidated general acceptance as a scientific tool by researchers all around the world, either in basic research or regulatory (Duval et al., 2017).

On the other hand, the progress in bioengineering technology has permitted to improve knowledge and application of cell culture, transforming a simple and standard biological toll into an innovative and dynamic assay for studying tissue and organ *in vitro* conditions.

In fact, as described by Pampaloni (Pampaloni et al., 2007), the third dimension bridges the gap between cell culture and live tissue; the authors pointed attention to the improvement that this model has made to the concept of cell-based assay. In fact, cell-cell and cell-ECM interactions build up a biological communication network able to guarantee the specificity and homeostasis of the cultured tissue. Another important milestone in the study and application of 3D structure, is the concept linked to the cellular context, that, as described by Bissell et al. (Bissell et al., 2003), represents the key point in the life cycle of the cells (proliferation, migration and apoptosis).

The concept and the importance of ECM are the basis of the three-dimensions application. In fact, ECM represents the environment where cells are targeted by multifactorial signals that move cells to different evolution/proliferation (Nikolova & Chavali, 2019). ECM can be constituted by several compounds that differ in physical and chemical construction. Usually, collagen is used in order to permit cellular adhesion to polyacrylamide or other gel. Hydrogel is another compound in 3D structure; modifications in the composition of hydrogel have been studied in order to investigate the relationship between cell spreading, proliferation and differentiation of mesenchymal stem cells (Chaudhuri et al., 2016). Another parameter linked to the interaction between cells and ECM, is represented by the forces exerted by ECM on cancer or immune migration. In fact, if in a 2D model, cells can grow on a flat area, in a 3D it should consider different spaces and the possibility of growing. Steinwachs J. et al. (Steinwachs et al., 2016) are pioneers in this field, and they have demonstrated that breast carcinoma cells migrate in similar ways even if the stiffness of the matrix is changed.

The knowledge and the application of 3D have grown in the last decades, and it has been almost clear that the difference between 2D and 3D is evident in different aspects: migrations, gene expression, morphology, etc.

Furthermore, the *in vitro* use of human cells can minimize the gap between the translation of the results into human medicine. In particular, there is a huge advantage in the evaluation of the data related to specific diseases or pathological mechanisms that could be miser understood if analyzed just with an animal model.

It is useful to remember the advantages/disadvantages associated with the use of three-dimensional *in vitro* models. Certainly, one of the strengths represented by these systems is to be able to mimic a biological “environment” in the laboratory and in a small and reportable space. From this point of view, reproducibility represents a significant advantage, together with the possibility of standardizing procedures/experiments. On the other hand, the lack of validated methods for different research environments considerably limits the possibility of using the different 3D methods in order to make such models as effectively substitutes for the use of the animal. Furthermore, one of the main limitations in using the 3D system is the difficulty of standardizing procedures, while 2D represents a historically consolidated model for different experimental approaches (Duval et al., 2017).

The three-dimensional models have provided a strong impetus in studies related to some branches of medicine and pharmacology. A good example of this is the progress made in cancer research. In fact, for breast carcinomas and bone tissue tumors, it was possible to consider new approaches, including therapeutic ones, thanks to the fact that the cellular interaction is better than the two dimensions, as well as the maintenance of morphological characteristics and histological of the cells of origin (Antoni et al., 2015).

Study of chemicals, particurally drugs, has found valuable help in the use of cells grown in three dimensions. This advantage is partly limited by the lack of reliability in the experiments which involve long evaluation times (Antoni et al., 2015).

2D cell cultures have aided the discovery of many biological and pathological processes, but are unable to mimic the complicated experience of microenvironmental cells in tissues (Costa et al., 2016; Lv et al., 2016). To predict the efficacy of a drug on a cell, a 3D culture model should mimic the tissue microenvironment in which cells can proliferate, aggregate and differentiate (Lv et al., 2016). Cells cultured in 3D showed different responses to drugs than cells cultured in 2D for several reasons. Differences in physical and physiological properties between 2D and 3D cultures mean that 2D cells are more susceptible to drug effects than 3D cells due to the fact that 2D cells are unable to maintain normal morphology as well as they can. Another reason why 2D cells are more sensitive to drugs than 3D cells is a cause of the difference in the organization of surface receptors on the cell. Third, cells grown in 2D are often all in the same cell stage while 3D cells are often found in different cell stages, just like cells *in vivo* (Lv et al., 2016; Langhans, 2018). In 3D cells, the difference in cell stage probably means that they are proliferating cells available in the outer region of the cell (Beekman et al., 2015). Many cell proliferation drugs are effective in promoting 3D cell culture (Langhans, 2018).

Metabolic profiling is used to demonstrate metabolic cooperation between different cell types and is becoming a popular technique in 3D culture models due to the accuracy of the results compared to *in vivo* cells (Tung et al., 2011). Previously, 2D culture models have been used to test cancer metabolism, but recent studies suggest that 3D culture models provide more information when testing the efficacy of new drugs (Russell et al., 2017).

Stem cells are commonly used in regenerative medicine and cell therapy. In clinical applications, however, 2D cell culture techniques have proved ineffective when using stem cells (Lv et al., 2016). This is because 2D culture is unable to accurately replicate the *in vivo* microenvironment of stem cells. Furthermore, MSCs often decrease in replicative capacity over time during 2D culture. However, when grown in spheroids, MSCs show a different morphology than 2D cultured MSCs (Cushing and Anseth, 2007) as well as having different gene expression patterns than those grown in 2D.

Through the use of spheroid cultures, MSC-based therapies have significantly improved. Organoids play an increasingly important role in the study of genetic diseases due to their ability to shape different regions of the body. For example, a rectal organoid was used to model cystic fibrosis to study the effects of modulatory compounds of the transmembrane conductance regulator, and another set of tubular organoids were used to model kidney disease where the microenvironment was found to play a key role in cyst formation (Dart, 2018). Furthermore, organoids have been shown to be useful models in the study of neurodegenerative diseases such as Alzheimer’s and Parkinson’s disease (Gurski et al., 2010; Dhaliwal, 2012). Brain organoids generated from pluripotent stem cells taken from Alzheimer’s patients when treated with β- and γ-secretase inhibitors, have shown promising therapeutic effects (Dhaliwal, 2012).

The development of perfusion and microfluidic systems led to what is known as the organ-on-a-chip model. These organ chips overcome many difficulties currently presented in ECM gel-grown spheroids and organoids (Sontheimer-Phelps et al., 2019). Although spheroids and organoids are useful ways to model many types of cancer, they have limitations due to the lack of tissue-to-tissue interfaces and organ-level structures (Sontheimer-Phelps et al., 2019). Organ chips are created using computer microchip fabrication and are populated with living cells that resemble organ-level physiology and pathophysiology *in vivo*. This is made possible by the *in vitro* construction of tissue and organ level structures that function like tissues and organs *in vivo* (Sontheimer-Phelps et al., 2019). Furthermore, these organs can provide accurate responses to many stimuli including drugs (Hassell et al., 2017). The idea of a human on a chip aims to examine normal human physiology within a microfluidic system by combining single-organ chips into a multi-organ chip design that allows organs to work together with each other just like the organs of the human body (Wang et al., 2020).

Understanding tumor characteristics by developing an accurate tumor model is the key to understanding the link between various types of cancer today. 3D cancer cells grown using 3D cell culture methods have won the spotlight in cancer cell biology research due to their innate ability to replicate the *in vivo* environment of a cancer cell *in vitro*. Aggregates of tumor cells are grown using 3D culture methods *via* suspension or gel embedding, mimicking tumor microenvironments *in vivo* (Lv et al., 2016). Multicellular tumor spheroids (MCTS) can be grown *via* static suspension, suspended drop methods, magnetic levitation, spinner bioreactor, rotational bioreactor, microfluidic system, and gel inclusion (Lv et al., 2016). These various methods allow for the replication of different microenvironments that can be found in specific types of tumors. Tumor-on-a-chip models have gained increasing popularity for the same reason as organ chips. A glioblastoma tumor was grown on a chip using C6 cells demonstrating that organ chip methods for drug testing in glioblastomas have high potential in future studies (Yu et al., 2019).

Wang et al., 2020 conducted a thorough investigation that aimed to mimic the progression of kidney cancer through a new 3D model of metastatic cancer cells.

Due to the recent success in treating melanoma skin cancer, researchers have begun to model melanoma cancer cells in 3D culture spheroids to target the molecular mechanisms that aid in resistance in current immunotherapy treatments (Müller and Kulms, 2018).

In an effort to understand how primary lung cancer progresses to metastatic lung cancer, one study used 3D cell culture techniques to track tumor cell migration (Xiong et al., 2019).

Furthermore, a new niche 3D model of the bone marrow was assembled to study the effects of a new class of engineered immune cells on primary myeloma cells. The 3D model outperformed 2D models with its ability to analyze specific homing as well as on-target and off-target effects. With the help of 3D cell culture, this niche 3D model of the bone marrow allows to study novel immunotherapies, mechanisms of resistance to therapy, and possible side effects of primary myeloma (Braham et al., 2018).

Both 2D and 3D cell culture techniques provide methods which are necessary for advancing research. 3D cell culture, however, has proven it has the potential to completely change the way in which new drug treatments are tested, diseases are modeled, stem cells are utilized, and organs are transplanted.

**FIGURE 2:**
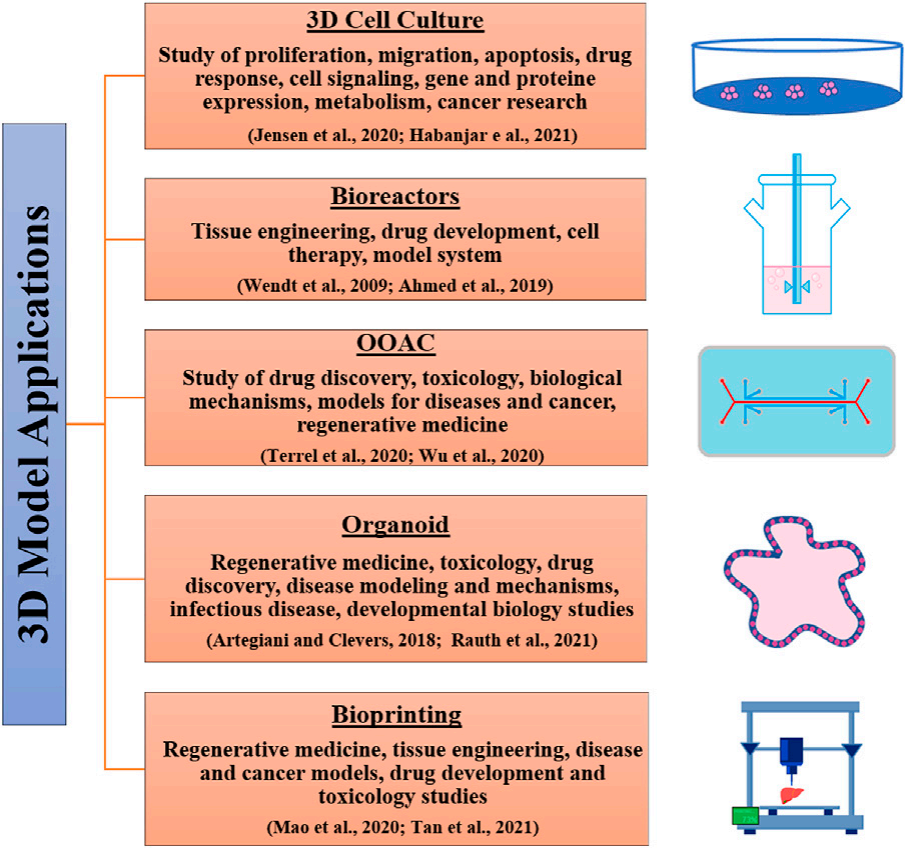
3D culture systems and their applications.

Table 1 and Figure 2 respectively, show the main differences between 2D and 3D cell culture systems and some fields of application of 3D models.

**TABLE 1:** Differences between 2D and 3D culture systems in the indicated parameters.

	2D cell	3D cell culture	References
*In vivo* imitation	This system do not mimic the *in vivo* microenvironment	3D cell culture replicate a higher number of *in vivo* features	Gurski et al. (2010)
Cell morphology	The cells grow flat and spread over the growth surface	Cell form aggregate/spheroid structure. Natural cellular structure preserved	Costa et al. (2016)
Cell-cell interaction	This system support cell-cell interaction	Models eplicate cell-cell and cell-matrix interactions	Sundarakrishnan et al. (2018), Lv et al. (2016)
Cell proliferation	Higher proliferation rate than in the natural environment	The proliferation and differentiation rates are specific to the cell line and also depend on the 3D system used	Xu et al. (2012), Lv et al. (2016)
Cell differentiation	Moderately and poorly differentiated	Well-differentiated	Lei and Schaffer (2013)
Cell survival	Cells are likely to be in the same stage of cell cycle due to being equally exposed to medium	Spheroids contain proliferating cells at the surface, whereas the interior possesses quiescent, hypoxic and necrotic cell	Tibbitt and Anseth (2009), Kim (2005)
Gene expression	Lower expression level compared to 3D model	More relevant expression level, similar to *in vivo* model	Birgersdotter et al. (2005)
Cost	Cheap	Expensive	Jensen and Teng (2020)

### Bioreactors

A bioreactor is a device capable of conveying the 3D technology applied to cell cultures, in a dynamic flow system that allows to apply mechanical/physical/chemical stimuli to a biological substrate and evaluate its effects. We can also say that bioreactors are the intermediate step between a 3D model and an organ-on-a-chip (OOAC) system. The concept and application of bioreactors is strongly linked to tissue engineering related to animal and plant cell culture (Wendt et al., 2009; Ahmed et al., 2019).

The cue to move from a static to a dynamic system started when it was seen how the methodology of inoculating the cells on a static 3D system did not guarantee a homogeneous growth of the cells themselves, preventing a correct design of the tissue architecture (Freed et al., 1994).

At the beginning of the use of these biological tools, their applications were essentially related to the adhesion of cell cultures on a scaffold capable of guaranteeing three-dimensional growth. This, together with the possibility of applying dynamic flow to allow an exchange of nutrients (enzymes, oxygen, etc.), makes this system able to recreate a microenvironment similar to that present in the *in vivo* model (Pei et al., 2002).

The main points of interest in the application and use of a bioreactor are the following:

i Conditions of inoculation of cells on the 3D matrix
ii Maintenance of the microenvironment.
iii Chemical/Physical conditions of the scaffold

The conditions of inoculation of the cells are one of the main parameters to be considered for the development of an experiment conducted with a 3D bioreactor. In particular, it is important to select some key points: concentration, type of cells used, methods of inoculation. In order to standardize a protocol of *inoculum*, it would be useful to apply a mathematical model. Li et al. developed a predictive computational model capable of optimizing the inoculation methods (cell density as a function of the porosity of the scaffold), in order to obtain the best result as a function of the parameters described above (Li et al., 2001).

Maintaining the microenvironment represents a critical point that can be kept under control in different ways. Tissue perfusion represents the key point linked to the vitality and correct architecture/functionality of the cells. This aspect is particularly important when entering the field of regenerative medicine, e.g. bone tissue reconstruction (Rauh et al., 2011). In addition, in this case, the possibility of first elaborating in theory mathematical models capable of optimizing the reference parameters would be very useful.

Bioreactors are limited by the lack of specific guidelines available in terms of flow rate/speed to use or volume of culture medium, as different cells have different cell culture requirements (Ismadi et al., 2014). Many bioreactor systems also do not incorporate the ability to non-invasively monitor the microenvironment in real time, which means important parameters such as oxygen, pH, temperature cannot be controlled. Therefore, the ability to monitor these parameters contributes to the refinement of the *in vitro* model (Kino-Oka et al., 2005).

Bioreactor systems should be chosen based on their specific application, some of which permit turbulent or laminar flow, others are more suited for suspension cultures or adherent cell types, and some bioreactors are necessary for larger scale culture.

Finally, a further aspect always linked to the perfusion of the tissue and the features of the scaffold to the mechanical/physical forces, to which a specific cell type is subjected *in vivo*, must be considered if the environment of the tissue under study is to be fully reproduced. From this point of view, the variables to be taken into consideration are many: pressure (blood vessels) (Altman et al., 2002), stretching (tendons, ligaments) (Kisiday et al., 2004), weight (bone) (Stiehler et al., 2009), tension (lung/alveolus) (Panoskaltsis-Mortari, 2015) etc. The optimization, monitoring and standardization of these parameters are the basis for the development of an *in vitro* model as close as possible to the animal model.

There are several types of bioreactors: spinner flasks, rotating wall vessels, perfusion bioreactors and microfluidic devices (Ahmed et al., 2019).

A spinner flask consists of a structure suitable for the growth of a biological substrate, in which the cells are suspended and subjected to magnetic forces that allow a continuous swirl of the culture medium. The scaffolds are located in a fixed position in the flask and the continuous movement of the medium allows the cells to adhere to the support, restoring the architecture of the original tissue. Another aspect favored by this system is that of cell differentiation and proliferation, as described by Stiehler (Stiehler et al., 2009). In this paper, the topic related to the application of the concepts of tissue bioengineering in the *in vitro* reconstruction of bone tissue is addressed. However, spinner flasks are thought to only permit the extracellular matrix production at the scaffold surface and mixing the media can create turbulent shear at the surfaces which can be unfavorable to cell growth and tissue formation (Gaspar et al., 2012). Cells maintained in the spinner flask system, can grow in batch mode. This is a closed type of cultivation system that does not allow for the addition of fresh medium or the removal of waste. This can limit the product yield, whilst overcoming the risks of contamination. This method is also limited both in scale and length of culture due to a build-up of metabolites and waste that occurs over time. Continuous cultures allow the removal of waste, but this exposes the culture to a maximum chance of contamination. It is possible to carry out medium changes and elimination of cellular residues, in a fed-batch system, but an external contamination can occur (Ahmed et al., 2019).

The rotating wall vessels (RWV) were developed by the National Aeronautics and Space Administration (NASA) to mimic the microgravity environment to which a biological system is subjected in space; with a particular regard to: structural, growth and regulatory processes (Schwarz et al., 1992; Gardner & Herbst-Kralovetz, 2016). Briefly, the RWV consists of a cylinder that rotates horizontally, it is equipped with an oxygenation system that guarantees correct cellular exchanges. The vessel system is filled with culture medium so that when the rotation begins, the cells remain in suspension, simulating the forces of gravity they are subjected to in the animal’s body (Navran, 2008). This device is able to reproduce a low fluid-shear that preserves the cells from potential damage caused by excessive agitation, facilitating the differentiation and proliferation of cell culture (Cherry & Papoutsakis, 1988). This cell cultivation system is applicable to different study approaches, from basic biology, to tissue engineering and to the study of infectious diseases, as described by Gardner (Gardner & Herbst-Kralovetz, 2016). Perfusion bioreactors and microfluidic devices are systems that ensure a constant flow of fresh or recirculating medium to the cell growing on scaffolds.

The possibility of creating a physiological environment in 3D allows to improve the exchanges of nutrients and oxygen between cells, improving differentiation, proliferation and construction of the original tissue. In Lembong J. et al., the development of a 3D fluidic system as a model to create a dynamic culture device for human Mesenchymal Stromal cells (hMScs), in order to evaluate the behavior and growth of cells an *in vitro* environment (Lembong et al., 2018). These systems are among the promising tools applied to bioengineering techniques. They will play a key role in the development of new approaches in the study of tissue regeneration and toxicology in general.

As mentioned above, bioreactors represent an important technological development to improve and innovate *in vitro* methods. The advantages deriving from the use of such systems have been seen above all in the cosmetic, toxicological and tissue engineering fields (Ma et al., 2021). In particular, the possibility of having three-dimensional “live” systems *in vitro*, allows the microenvironment and the organ/tissue to be analyzed in a completely similar way to the animal organism.

The ability to cultivate tissues *in vitro* using bioreactors has been considered a milestone in regenerative medicine as scaffolds not only function as a support for living cells or as a vehicle for growth factors, but also aid in the process of formation and/or regeneration of cells and tissues. However, the development of biomimetic scaffolds and bioreactors is still very challenging and requires specific improvements to enable the clinical translation of these technologies into regenerative medicine. Furthermore, this type of technology can be used to support the production or fabrication of cellular products in a clinically and commercially feasible way, for example by aiding *ex vivo* cell therapies, such as patient-specific approaches (Castro et al., 2020).

The challenges related to the development of bioreactors and scaffolds need to consider the following conditions: 1) considering the vascularization of tissues *in vitro* should be a priority in these experiments, which would allow them to be adequately prepared for *in vivo* vascularization, at the time of plant; 2) include an inflammatory environment along with the growing tissue for optimal tissue development, since inflammation is an essential component of the normal mammalian host tissue response and must be present for a biomimetic approach; 3) constant monitoring of the bioreactor environment and tissue development using advanced imaging and sensing modalities is important for monitoring cell fate and tissue development in the complex 3D environment.

This recent field of engineering is not only providing interesting results, but has also been shown to have ample room for improvement by assembling different types of stimulus mechanisms in order to continue improving these biomimetic apparatuses in the coming years, allowing new and essential insights into tissue regeneration.

### Organs-On-A-Chip

The term “Organ-on-a-Chip” (OOAC) refers to a biotechnology that currently represents one of the most promising and exciting developments related to the possibility of combining biology and engineering. Through the use of state-of-the-art manufacturing techniques, such as photo and soft-lithography, the possibility of engineering biomimetic platforms on chips has recently been demonstrated (Huh et al., 2011).

These biochips have shown enormous potential in cultivating living cells in a controlled environment characterized by a micrometric scale, therefore comparable to that perception by cells in our body. These engineered biomimetic microenvironments ultimately allow replicating *in vitro* (i.e. in the laboratory) structures and functions characterizing native organs and tissues with a precision not obtainable with traditional technologies.

The biological “micro-constructs” obtainable through this approach are intended with the ultimate goal of the minimal functional unit characterizing a specific organ (for example, a portion of the alveolus or a renal nephron). Despite the apparent simplification, these platforms are equipped with effectively replicate the complexity necessary to obtain physiological-like functional responses *in vitro*. Thanks to the demonstrated ability to integrate the peculiar functions of the different tissues, their respective interfaces and, at the same time, to recreate the most chemical/physical characteristics relevant to the specific cellular microenvironment.

This model is particularly useful in pre-clinical approach during the study of drug development, for the screening of the compounds and in toxicological evaluation. The routine process provides for the use of cell culture and *in vivo* models (Terrell et al., 2020).

The ability to control the flow of air or the culture medium in microchannels, combined with the ability to subject cells and three-dimensional (3D) constructs to dynamic stimulation, has allowed, for example, to obtain systems that keep cells alive for long periods of time outside the living organism, promoting its differentiation into complex functions, up to obtaining *in vitro* physiology and pathophysiology of organs and tissues.

OOAC systems therefore offer unprecedented possibilities to recreate functional models of healthy human tissues *in vitro* and at the same time to model pathological states or systemic reactions to certain drugs. For this reason, these tools may be a thoughtful approach to technology that can revolutionize healthcare (Marsano et al., 2016). In particular, it is a widespread opinion that the growing development of OOAC will have a huge impact on the research and development sectors of new drugs and molecules, allowing to analyze the pharmacokinetic and pharmacodynamic effects within engineered platforms.

These biochips are potential and promising candidates to replace the animal models that are now the Golden Standard in multiple areas of biomedical research. Animal models currently used in biomedical research and development are in fact considered an expensive and ethically questionable approach, as well as often failing in predicting human responses due to intrinsic interspecies diversity (Knudsen et al., 2019). This current inability of the models available today to effectively replicate human physiology is in fact one of the main causes of the partial ineffectiveness of the development process of new drugs, often very expensive and extremely long (up to 10 years).

Driven by the growing need to develop more representative models of the human physiological response, and at the same time by the request to reduce animal experimentation to the bare minimum in compliance with the 3Rs principle, the development of OOAC is becoming more widespread. Its growth is increasingly accelerated thanks to the integration of multidisciplinary knowledge of biology, engineering, chemistry, materials, and physics (Terrell et al., 2020).

From this point of view, OOAC has, potentially, an enormous variability in use. A particular aspect is represented by the regenerative medicine and personalized medicine, which is the science of the near future. As described by Arrigoni et al., the possibility to associate the application of 3D bioprinting with the microfluidic organ-on-chip is the step to the medicine of the next generation (Arrigoni et al., 2017). In this paper, the authors highlight the progress of this kind of approach, stressing the importance of these devices as a functional model to improve the bone graft model and the future potential application.

The feasibility of setting up a research using the organ-on-a-chip system is strongly connected with the physical/chemical/mechanical knowledge of the materials that support the cells used (Terrell et al., 2020). There are several possibilities for setting up a 3D model. The step beyond, is the integration of this microfluidic system into an organs-on-chip. In Terrell at al. are described different approaches useful for an integrated approach between the two methods. For example, Skardal et al. proposed an *in situ* 3D liver model as a device for drug development (Skardal et al., 2012). They demonstrated the vitality for at least 7 days of HEPG2 cells using a hydrogel added with a native ECM. The whole system is able to support the photopolymerization with zonal discrimination and it is useful in order to obtain an *in situ* 3D model adapted to an organ-on-a-chip.

The liver represents one of the models most widely studied for the evaluation of the safety of different chemical compounds, including cosmetics. Strong efforts have been made by chemical industries to develop useful devices for the evaluation of substances used in cosmetics. The advent of microfluidic has allowed the development of the liver-on-chip model as a response to the ban of *in vivo* model. Unfortunately, this device still shows many application limitations, especially regarding the accuracy of the data obtained for the physiology and toxicokinetic of the compounds studied (Ehrlich et al., 2019).

Given the advances in OOAC technology, microfluidic chips certainly provide favorable support for the development of OOAC. Numerous OOACs have been designed and prepared. A range of human organs was investigated with the ultimate goal of integrating numerous organs into a single chip and building a more complex multi-organ chip model. Although this technology has developed rapidly, the theory of a multi-organ chip model still remains a long way off. This is due to various factors such as the need to identify suitable materials or the cost of experimental production and implementation, which is relatively expensive, which does not favor the widespread use of organ chips. Even the collection of samples on the chip can interfere with its operation, resulting in variations in the concentration of various metabolites. Just universal cell culture media suitable for all organs is needed. Above all, as the number of organs on the chip increases, functionality becomes more complex and the data generated carries artifacts and untranslatable risks. *In vitro* biomarker analysis, following long-term repeated dosing, may not fully reflect *in vivo* studies. All these problems still remain a crucial point to be taken into consideration in the approach to these methods.

### Organoids

Organoids are 3D *in vitro* cellular structures that display architectures and functionalities similar to *in vivo* organs and that develop from stem cells or organ-specific progenitors through a self-organization process (Clevers, 2016). Organoids are classified into stem cells and tissue organoids (Huch & Koo, 2015).

Stem cell organoids can be generated from pluripotent Embryonic Stem (ES) cells and their synthetic induced Pluripotent Stem (iPS) cell counterparts and organ-restricted adult Stem Cells (aSCs) (Lancaster & Knoblich, 2014). Both approaches exploit the seemingly infinite expansion potential of normal stem cells in culture.

Tissue organoids refer to mesenchymal cells and mainly apply to epithelial cells for their ability to self-organize into tissue structures also thanks to the development of growth factors that mimic the various organ stem cell niches.

To date, several *in vitro* organoids have been established to resemble various tissues, including functional organoids for thyroid (Antonica et al., 2012), pancreas (Greggio et al., 2013), liver (Takebe et al., 2013), stomach (Stange et al., 2013), intestine (Spence et al., 2011), vascularized heart patch (Stevens et al., 2009), cerebral cortex (Lancaster et al., 2013), thymus (Bredenkamp et al., 2014), kidney (Wang et al., 2020), lung (Lee et al., 2014), and retina (Nakano et al., 2012). The wide range of tissue types, the long-term expansion capacity, and the physiological 3D architecture of organoids make them a powerful new technology for many biological and clinical applications, providing highly informative and complementary approaches to established 2D culture methods and animal model systems.

Organoids are suitable for infectious disease studies. For example, brain organoids were used to study the impact of Zika virus (ZIKV) on human brain development and the mechanistic link between ZIKV infection and microcephaly (Wen et al., 2017). Intestinal organoids have been used to study host-pathogen interactions for human enteric viruses, such as Rotavirus (Yin et al., 2015) and Norovirus (Ettayebi et al., 2016). *Helicobacter pylori* interactions with the stomach epithelium were also investigated by directly injecting the bacterium into the lumen of gastric organoids. The ability of cells to differentiate into different stomach lines has shown that gastric gland cells exhibit the highest inflammatory response to *Helicobacter pylori* (Bartfeld et al., 2015). More recently, due to the strong association with COVID-19 and diabetes (Zhu et al., 2020), hepatic organoids derived from human Pluripotent Stem Cells (hPSC), which were found to be highly permissive to SARS-CoV-2 infection, have been used to systematically explore the viral tropism of SARS-CoV-2 and cellular responses to infection (Yang et al., 2020).

Organoids are increasingly being used to model human genetic diseases. Organoids derived from patient biopsies or genetically engineered wild-type organoids appear to represent a winning approach to study the effect of lethal mutations during development or in early life. In 2013 Dekkers and others obtained intestinal organoids from rectal biopsies of a series of patients with cystic fibrosis (CF). They developed a test in which healthy organoids respond to Forskolin treatment by rapid swelling, while this effect is greatly reduced in CF organoids. This test has proven very reliable for predicting responses to Cystic Fibrosis Transmembrane conductance Regulator (CFTR) and has become the first personalized organoid medicine application for cystic fibrosis patients in the Netherlands (Dekkers et al., 2013). Intestinal organoids with pathogenic mutations in the TTC7A domain have been derived from multiple patients with intestinal atresia (Bigorgne et al., 2014). These organoids have shown that TTC7A deficiency leads to a reversal of apicobasal polarity in the intestinal epithelium that can be recovered by inhibiting RhoA kinase signaling (Bigorgne et al., 2014). The intestine is not the only organ from which patient-derived organoids have been isolated. For example, hepatic organoids derived from patients with 1-antitrypsin deficiency (A1AT) and Alagille syndrome (Huch et al., 2015) reproduced the defects observed *in vivo* (Andersson et al., 2018). These studies show not only how organoids can faithfully summarize disease characteristics in a human *in vitro* model, but are a valuable resource for both basic research and therapy development.

Patient-derived iPSC-generated brain organoids have been used to study human microcephaly, a genetic disorder caused by a mutation in CDK5RAP2 (Lancaster et al., 2013). Forebrain organoids have been used to investigate a genetic condition causing lissencephaly, which shows defects in progenitors and Wnt signaling (Bershteyn et al., 2017). *Raja* et al. developed iPSC-derived brain organoids from patients with Alzheimer’s disease (AD), the most common type of dementia, characterized by the extracellular deposition of misfolded amyloid-β containing plaques and intracellular neurofibrillary tangles. The developed model treated with *β* and γ-secretase inhibitors can significantly reduce amiloid-β and tau pathology, demonstrating the potential of using human brain organoids for drug discovery in AD (Raja et al., 2016).

Organoid technology in recent years has opened an unprecedented approach also for the study of human tumors *in vitro*. Patient-derived cancer organoids, which retain both the heterogeneity and genetic characteristics of their original tumor tissues, could be widely used in the future of personalized cancer medicine. Cancer lines have been successfully established from various cancers, including colorectal, pancreatic, liver, breast, prostate, brain and bladder cancers, from both primary and metastatic cancers (Boj et al., 2015; Fujii et al., 2016; Gao et al., 2014; Huang et al., 2015; Hubert et al., 2016; Lee et al., 2018; Sachs et al., 2018; Seino et al., 2018; van de Wetering et al., 2015; Weeber et al., 2015). Organoids are useful for studying the role of mutational processes in tumorigenesis (Stratton et al., 2009). Gene editing technologies, such as CRISPR-Cas9, coupled with the use of healthy organoids has led to a better understanding of organ-specific mutagenic processes (Matano et al., 2015) resulting from the accumulation of key mutations during malignancy transformation (Drost et al., 2017). For example, the introduction of a combination of driver mutations in KRAS, APC, TP53, and SMAD4 was used to generate colorectal cancer (CRC) progression models (Matano et al., 2015). In another study, oncogenic mutations in CDKN2A, KRAS, TP53, and SMAD4 introduced into the human pancreas organoids transformed normal cells into cancerous cells (Drost et al., 2017). Cancer organoids have also been used to model metastatic processes, in particular to study the different invasion processes. Microscopic observations showed how, in breast cancer organoids, specialized tumor cells expressing K14 and p63, with invasive phenotype, extended multicellular filaments of tumor cells into the extracellular matrix (K. J. Cheung et al., 2013). Or that cathepsin B led to collective invasion in salivary cystic adenoid carcinoma (Wu et al., 2019) the inhibition of Rho-associated protein kinase 2 (ROCK2) associated with the initiation of collective invasion in colorectal adenocarcinoma (Libanje et al., 2019), and the loss of heat shock factor 2 (HSF2) correlated with collective invasion in prostate cancer (Björk et al., 2016).

Currently, organ replacement therapy of diseased or damaged tissues relies largely on allogeneic transplantation. Recent organoid technology suggests that patient-derived organoids could be considered as alternative treatment strategies to organ transplantation. Yui et al. demonstrated that mouse colon organoids could be expanded and grafted into the damaged mouse colon and form functional crypt units (Yui et al., 2012). Human PSC-derived intestinal organoids were subsequently transplanted into mice under renal capsule and exhibited a structure with permeability and peptide uptake properties, highlighting translational potential for the treatment of short bowel syndrome and other gastrointestinal diseases (Watson et al., 2014). Adult mouse liver organoids have been shown to resolve liver failure and prolong survival rate after transplantation in a mouse model of type I tyrosinemia (Hu et al., 2018). Similarly, PSC-derived hepatic organoids were able to rescue acute liver failure and restore liver function (Nie et al., 2018). Retinal tissues generated from human ESC-derived organoids also survive, mature and demonstrate a degree of integration with the host tissue when transplanted into rat and primate models of retinal degeneration (Shirai et al., 2016). Furthermore, organoids could potentially be combined with gene correction as an alternative approach for the treatment of monogenic inherited degenerative diseases. For example, gene correction of CFTR mutation in patient -derived organoids (PDOs) using CRISPR/Cas9 gene editing could repair CFTR function (Schwank et al., 2013). It will be important to explore the therapeutic potential of other degenerative diseases associated with a single gene. While the potential of organoid applications in regenerative medicine is promising and exciting, it is important to address the safety, ethical, and legal issues before moving to the clinic.

Cultures of human organoids have several potential advantages, particularly over animal models organoids provide faster and more robust results, are more easily accessible, and provide both a more accurate representation of human tissue and more material to work with than animal models. Cultivating organoids derived from human stem cells can bridge the remaining gaps between animal and human models, mainly because the starting material for organoid culture is a human stem cell. One of the most interesting perspectives in basic organoid research is the ability to study human development (and disease) without tissue accessibility constraints. The path to a wide-ranging translation of organoid technology into real-life preclinical and clinical applications is considerably more complicated. However, studies are emerging that demonstrate the potential of organoids in settings of personalized medicine, drug discovery, regenerative medicine and gene therapy, suggesting that the more widespread adoption of organoids in these fields could become a reality (Fowler et al., 2019; Lancaster MA, Huch, M. 2019).

Much has already been achieved in revealing the incredible level of self-organization that stem cells can show when grown under the appropriate 3D conditions and in expanding the list of organoid types. However, there are still some limitations. The main challenges include regulating self-organization to generate organoids that develop with physiologically relevant shapes and sizes; extend the lifespan of organoids to create mature, functional tissues that achieve homeostasis. In the study of tumors, for example, the stromal component, as well as the fibroblasts, the surrounding endothelial cells, the immune cells and the ECM are essential for reconstituting the tumor microenvironment; the lack of this microenvironment could compromise the application to predict the clinical outcome.

Overcoming these challenges, thanks to a multidisciplinary approach, can lead to particularly impactful results.

### Bioprinting

Bioprinting is the ultimate and most progressive step of engineering applied to cell culture. As described by Groll et al., 2018 the first concept related to this topic is the terms “bioink” and “biopaper”; in fact, at the beginning of organ printing, this concept was linked to hydrogel as paper and cell culture as ink. In these last year, biotechnology innovation permitted to arrive to a new definition of bioink, as a formulation constructed by different kinds of cells, or biological material, or a mixture of these biological compounds.

In recent years, advances in tissue engineering, cell biology and materials sciences have made it possible for 3D bioprinting to create functioning tissue or organ grafts with their natural microenvironments and autologous cell architectures for transplant applications.

This technology, based on living cell cultures, biocompatible materials and digital support tools, enables the layer-by-layer arrangement of biomaterials, biochemicals and living cells with accurate spatial control, thus mimicking the systemic complexities of conditions physiological or pathological (Guillotin et al., 2010; Moon et al., 2010).

The generation of functionally viable tissues-in-a-dish requires a specific niche and microarchitecture that should provide structural and mechanical support, sufficient nutrient supply, the cell types required and the ability to remodel and integrate with the host once implanted (Perez-Castillejos, 2010; Schubert et al., 2014).

3D bioprinters create cellular models within defined spaces while simultaneously preserving cell function and viability (Wüst et al., 2011). This process usually requires an important component, “bio-ink” or material that mimics an ECM environment to support cell adhesion, proliferation and differentiation (Murphy & Atala, 2014). Normally, the cells to be printed are dispersed throughout the bioink, which is often generated by a hydrogel (Munaz et al., 2016).

Although 3D bioprinting has become the most promising method in tissue engineering due to its ability to control the geometry and amount of biomaterial used in construct fabrication, there are still some questions to be explored, such as cell viability and vascularity of printed tissues that they must be organized in larger and more versatile tissues or organs.

3D bioprinting is needed to accurately deposit cells, biomaterials and biomolecules layer by layer from computer-aided equipment and software, which have been possibly built by integrating modern scientific and technological knowledge, including cell biology, engineering, science of materials and information technology (Jessop et al., 2017).

Various 3D bioprinting platforms can already generate different types of tissues.

3D stem cell bioprinting approaches can have enormous implications in regenerative medicine, for the modeling and treatment of heart disease, and heart failure, as well as for toxicology studies and personalized drug testing (Ryu et al., 2015). Engineered myocardial grafts are currently in preclinical studies and may 1 day serve as cost-effective and efficient solutions for myocardial infarction. 3D bioprinting also has potential in valvular disease, as the ability to accurately reconstruct native heart valves has enormous clinical implications, including surgical planning processes and regenerative medicine (Cheung et al., 2015).

Bioprinted skeletal muscle tissue proposes promising methods for the development of novel bioengineering microdevices, such as motors, actuators, heart pumps and biosensors, or it can be used in muscle exercise studies. Furthermore, musculoskeletal tissue bioprinting can be used to improve the design of treatment materials for musculoskeletal disease and trauma, as well as for regenerative medicine applications (Gao & Cui, 2016).

3D bioprinting of neural tissue stem cells will facilitate research into neural development, function and disease processes, as well as translational drug screening *in vitro* (Gu et al., 2016). There are also possible applications in patient-specific neural tissue engineering for central nervous system (CNS) tissue replacement following acute traumatic injury and chronic degenerative disease (Hsieh & Hsu, 2015; Gu et al., 2016). Resistance to chemotherapy drugs is a huge challenge in brain tumors, largely due to brain tumor stem cells, and therefore the use of *in vitro* constructs that recapitulate the microenvironment of native tumor tissue has enormous potential to improve the current therapeutic regimens and developing new treatments (Dai et al., 2016). As well as the replication of patients’ tumors *in vitro*, it may allow for the personalization of therapies and individualized tumor tests for drug resistance and susceptibility (Dai et al., 2016).

Engineered skin tissue has significant clinical implications. Engineered skin grafts have been evaluated in preclinical studies and offer much promise in regenerating and repairing damaged skin tissue. Bioprinting technologies have particularly important implications for skin tissue regeneration, as this technique provides an accurate and fast way to deliver tissue grafts directly to the wound site, as has been demonstrated in multiple experimental models (Skardal et al., 2012).

Bone tissue is the most common type of hard tissue considered in the context of 3D bioprinting. Bone defects and injuries resulting from aging, trauma, infection, disease or failed arthroplasty often require tissue reconstruction using a metal graft or implants (Ashman & Phillips, 2013). Bioprinting is expected to be a powerful tool for bone tissue engineering, as it can build 3D constructs to reproduce bone microstructure. In fact, 3D printed scaffolds for bone regeneration, thanks to their 3D structure with desirable porosity and mechanical properties that can mimic natural trabecular bone, represent a promising alternative to conventionally used devices. Additionally, bioprinting has the potential to improve clinical outcomes of bone repair as it may overcome some current side effects of bone grafting. Furthermore, pre-vascularization strategies have been developed that aim to resemble the highly vascularized nature of bone using vascular endothelial growth factors (VEGF) and endothelial cells (Daly et al., 2018).

3D bioprinting is emerging as a promising technology for fabricating complex tissue constructs with tailored biological components and mechanical properties. Recent advances have enabled scientists to precisely position materials and cells to build functional *in vitro* tissue models for disease modeling and drug screening. In this regard, Yi et al. (Yi et al., 2019), highlighted that bioprinted reconstituted glioblastoma tumors consisting of patient-derived tumor cells, vascular endothelial cells and extracellular matrix decellularized by brain tissue in a concentric ring structure cancer-compartmented stroma, summarizes the structural, biochemical and biophysical properties of native tumors. They also showed that glioblastoma-on-a-chip reproduces clinically observed patient-specific resistances to treatment with concomitant chemoradiotherapy and temozolomide and that the model can be used to determine drug combinations associated with superior tumor killing. The patient-specific tumor model on a chip could be useful for identifying effective treatments for patients with glioblastoma resistant to standard first-line treatment.

Despite significant advances in skin 3D cell printing for regenerative medicine, there are still few disease models that show pathological processes found in native skin. In this regard, (Kim et al., 2020; Kim et al., 2021), recently modeled diseased 3D skin tissue with pathophysiological signs of type 2 diabetes *in vitro* based on the 3D cell printing technique. By stimulating the epidermal-dermal intercellular crosstalk found in native skin, it was hypothesized that normal keratinocytes would differentiate as diabetic epidermis when interacting with the diabetic dermal compartment. To prove this, a novel wounded skin model was successfully devised during tissue maturation *in vitro*. Using the versatility of 3D cell printing, the structural similarities and diabetic properties of the model were further augmented by addition of perfusible vascularized diabetic hypodermis. Insulin resistance, adipocyte hypertrophy, inflammatory reactions, and vascular dysfunction, as the typical hallmarks in diabetes, were found under hyperglycemia. Finally, the feasibility of this new disease model for drug development was successfully demonstrated through application of different test drugs. This study provides a pioneering step towards 3D cell printing-based *in vitro* skin disease modeling.

Lee et al. (Lee et al., 2020) developed on-chip 3D liver fibrosis with three types of liver cells (hepatocytes, activated stellate cells, and endothelial cells) using a novel cell printing technique with gelatin bio-inks, which were used to deliver each non-parenchymal liver cell type as a multilayer construct. Gene expression specific for liver fibrosis, collagen accumulation, cell apoptosis, and decreased liver function caused by activated stellate cells were also evaluated. Additionally, some chemicals were added to 3D liver fibrosis on a chip to examine the downregulation of activated hepatic stellate cells. The developed 3D liver fibrosis-on-a-chip could be used as a potential *in vitro* disease model and drugs response system.

While 3D bioprinting is advancing at a commendable pace with continuous updates and improvements to existing modes, there still remain several challenges that need to be overcome.

One of these concerns the limit of some basic bio-inks for the definition of the tissue architecture is necessary to restore organ function after printing. While naturally derived hydrogel-based bio-inks promote cell growth, synthetic hydrogels are mechanically robust. Therefore, hybrid bioinks should be designed to combine all of these aspects. Furthermore, the bioprinting process itself needs to be more compatible with the cells. Stress applied to cells during the printing process is detrimental to cell growth and could even alter gene expression profiles. Stem cells, such as iPSCs, are sensitive to such physical forces and usually do not survive the printing process.

Furthermore, the problem of the vascularization of the bioprinted constructs for a correct exchange of nutrients, as well as the integration of the printed vascularity with the host vascularization after organ implantation, is another important obstacle. Overall, 3D bioprinting is a rapidly evolving research field with immense challenges, but huge potential to revolutionize modern medicine and healthcare.

## Conclusion

The animal model still represents the gold standard for numerous studies, in particular for regulatory aspect and, in general, for the safety/efficacy evaluation of drugs and biologicals. Neithertheless, the efforts and the new technology developed as *in vitro* method, can and should demonstrate the path for the science of the future.

A multidisciplinary approach involving different non-animal based methods represents the goal of a modern concept of research.

In particular, the possibility to improve the use of cell culture linked to bioengineering techniques allows the development of a different concept of *in vitro* assay, with an improvement of 3Rs application as requested by European Directive.

As described in this review, different ways to investigate the use of cell culture in mimicking the *in vivo* model have been developed. For this reason, it is important to fully understand the potential use of these methods and their contribution to the development of a different and alternative pattern of research.

Form this point of view, it is important not to forget that the correct application of these new methods is also strictly related to their limitations. In fact, as already discussed, it is not yet possible to reproduce in a complete and efficient way happens in an *in vivo* model.

The strategy for a paradigm shift of the experimental design lies in different aspects, one for all is the sharing of information and a correct application of 3Rs principle.
